# The Translation and Preliminary Psychometric Validation of the Ghosting Questionnaire in Urdu

**DOI:** 10.3390/ejihpe14030037

**Published:** 2024-03-01

**Authors:** Waqar Husain, Asma Sadiqa, Eman Zahid, Fatima Idrees, Achraf Ammar, Zahra Saif, Khaled Trabelsi, Seithikurippu R. Pandi-Perumal, Mary V. Seeman, Haitham Jahrami

**Affiliations:** 1 Department of Humanities, COMSATS University Islamabad, Islamabad Campus, Park Road, Islamabad 45550, Pakistan; drsukoon@gmail.com (W.H.); sadiqa.asma1@gmail.com (A.S.); emanzahid835@gmail.com (E.Z.); fatimaidrees480@gmail.com (F.I.); 2 Department of Training and Movement Science, Institute of Sport Science, Johannes Gutenberg-University Mainz, 55099 Mainz, Germany; acammar@uni-mainz.de; 3 Research Laboratory, Molecular Bases of Human Pathology, LR19ES13, Faculty of Medicine of Sfax, University of Sfax, Sfax 3000, Tunisia; 4 Governmental Hospitals, Manama P.O. Box 12, Bahrain; zahra-saif@outlook.com; 5 High Institute of Sport and Physical Education of Sfax, University of Sfax, Sfax 3000, Tunisia; trabelsikhaled@gmail.com; 6 Research Laboratory: Education, Motricity, Sport and Health, EM2S, LR19JS01, University of Sfax, Sfax 3000, Tunisia; 7 Division of Research and Development, Lovely Professional University, Phagwara 144411, Punjab, India; pandiperumal2023@gmail.com; 8 Saveetha Medical College and Hospitals, Saveetha Institute of Medical and Technical Sciences, Saveetha University, Chennai 600077, Tamil Nadu, India; 9 Department of Psychiatry, University of Toronto, Toronto, ON M5R 0A3, Canada; mary.seeman@utoronto.ca; 10 Department of Psychiatry, College of Medicine and Medical Sciences, Arabian Gulf University, Manama P.O. Box 26671, Bahrain

**Keywords:** assessment, ghosting, psychometrics, scale, Urdu

## Abstract

Background: “Ghosting” refers to the practice of abruptly cutting off all contact with a person with whom you have been in constant correspondence. The break comes without warning and without understandable provocation. The term most commonly applies to online romantic relationships. The motives for and effects of ghosting have been studied, and validated research questionnaires have been developed; however, there are no such questionnaires available for Urdu speakers. The purpose of this study was to adapt the “Ghosting Questionnaire (GQ)” for use in Pakistan and India, two of the world’s most populous countries—a process that involves translation, adaptation, and validation. Methods: The study’s methodology involved translating the GQ into Urdu using both forward and backward translation techniques. Convergent validity, test–retest reliability, internal consistency, confirmatory factor analysis, and goodness of fit were all components of the psychometric analyses. Conclusions: The Urdu version of the GQ demonstrated a good internal consistency, with the Cronbach’s alpha and McDonald’s omega both exceeding 0.90. It also showed a high test–retest reliability—(0.96). The one-factor structure was confirmed by the confirmatory factor analysis, which agreed with the original English version of the GQ.

## 1. Introduction

Ending an intimate relationship leads to heartbreak for many people and is emotionally difficult to initiate. The end can be gradual or abrupt; it can involve many troubling discussions and in-depth, uncomfortable, explanations. This means that many individuals consider it far easier to avoid face-to-face meetings and, instead, simply stop responding [1]. Ghosting is a form of avoiding a person with whom one was corresponding by abruptly (or gradually) ending all communication with no explanation [2,3]. Common examples of ghosting include “unfriending” or “unmatching” on social media or failing to respond to phone calls, emails, or text messages [1,4]. According to new research based on interviews with ghosters and ghostees [5,6], ghosting can also occur in platonic friendships. This contradicts earlier claims of researchers such as LeFebvre and Koessler [7,8], who restricted the term to romantic relationships.

Ghosting has arisen due to changes in interpersonal mores and personal communication over time [9]. Face-to-face meetings have given way to a modern way of communicating through media [7], particularly social media [6,10]. Due to technological advancements, people can now instantly ‘talk’ with anyone anywhere in the world, regardless of temporal differences, geographic distance, or even language. With the advent of online dating apps and websites, long-distance relationships can consolidate through texting and video chatting. Conventional methods of introduction via family or friends, with preliminaries such as an exchange of notes or phone calls, followed by a succession of chaperoned meetings, have fallen by the wayside. Getting to know one another through mobile applications is now the most popular way to seek and find romance. The explosion of social media and the growing number of online communication apps have made ghosting possible and omnipresent [11,12,13]. A new phenomenon known as “online vigilance” has emerged. This refers to increased preoccupation and involvement with what is going on online, the constant monitoring of online events on dating apps, for instance, and a tendency to respond to them. This means that in-person interactions have decreased, spurred recently not only by technology, but also by the COVID-19 pandemic [14,15]. The perceived freedom of dating-at-a-distance has facilitated ghosting without guilt at the thought that a close person’s feelings have been hurt. The widespread use of mobile devices and social media has made “ghosting” the go-to method for ending a relationship without the discomfort of a face-to-face confrontation [16].

While ghosting can technically occur in any social interaction, it is far more prevalent and problematic in online communication spaces [11,12,13]. In face-to-face interactions, one cannot avoid signs of disappointment, hurt, anger, or demands for explanation. This makes it hard for one party to abruptly break off [11,12,13]. Online spaces allow for distance and a certain amount of anonymity, which makes ghosting an easier option [6,12,13]. This distinction between face-to-face confrontation and rejection at-a-distance is important to understand, as the rise of ghosting is a direct result of the expansion of online messaging, online dating, and online social platforms [15]. The lack of in-person accountability online enables ghosting behaviors, and such behaviors have psychological consequences [15], which is why ghosting has emerged as an interesting area of study in psychology.

Recent research has revealed that individuals with “dark triad” traits, characterized by narcissism, Machiavellianism, and psychopathy, are the most likely to avoid the awkward and emotionally draining conversations associated with ending a relationship [1,16,17]. These individuals have little empathy for the people they hurt; instead, they are driven by the need to avoid distress for themselves [3]. Individuals with narcissistic personalities prefer to avoid confrontations where they may be accused of being disloyal or self-serving [4]. Machiavellians, who exhibit traits such as dishonesty and duplicity, may engage in multiple relationships simultaneously, and, after a time, drop those of lesser significance [1,3,17]. A fundamental lack of empathy for others facilitates ghosting [1]. According to attachment theory, an infant’s early inability to bond with parental figures lies at the root of attachment avoidance in adulthood; ghosting can be understood in this context [18]. Avoidance of potentially stressful situations is considered to be a mechanism of defense that serves to shore up a fragile self-image [8].

The investigation of ghosting behavior necessitates a thorough exploration of its psychometric dimensions, employing reliable and validated tools. To address this need, Jahrami and associates (2023) recently introduced the Ghosting Questionnaire (GQ), an eight-item English-language questionnaire, rooted in the Shannon–Weaver communication model (Shannon, 1949). The GQ is constructed around six crucial components associated with ghosting, namely: sender, encoder, channel, noise, decoder, and receiver. According to this model, the encoder transforms a sender’s message, making it transmissible. The path that the encoded message takes during transmission is referred to as the channel, and noise is any unwanted interference that could distort or otherwise affect the message’s accuracy or clarity. The receiver is the person who reads the message after it has been deciphered by a decoder who transforms it back into its original form. Many things can go wrong along this pathway. For example, a sender sends a message through a mobile phone network, expecting a reply. When there is no response, the sender does not, at first, know whether a fault occurred somewhere across the communication chain or whether the receiver chose to disregard it, delete it, or plans to respond later. When further messages fail to elicit a response, the recipient realizes that they are the victim of ghosting. Such a realization—that one has been rejected without any understandable reason—has negative implications for mental health. This study endeavors to contribute to the wider identification of these implications by translating the GQ into Urdu and validating this translation, thus facilitating future research on ghosting within the cultural context of South Asia. This translation aims to significantly enhance the opportunities for Pakistani and Indian researchers to explore the phenomenon of ghosting within their vast population. Considering that India is the second-most populous country and Pakistan is the fifth-most populous country globally, this study is relevant for understanding the prevalence and consequences of ghosting for a large portion of the globe. The hypothesis of this study is that the translated Urdu version of the GQ will demonstrate an adequate reliability and validity for measuring ghosting behaviors and relevant emotional responses within Urdu-speaking populations. We predict that the factor structure and psychometric properties of the translated questionnaire will be comparable to those of the original English version. This will test the socio-cultural utility of the GQ instrument.

## 2. Materials and Methods

### 2.1. The Process of Translating the GQ into Urdu

The translation and back-translation of the GQ involved five essential steps. First, two expert translators fluent in both English and Urdu were assigned the task of making the survey accessible to Urdu speakers while capturing the precise meaning of each item. The two translators first agreed on the accuracy and clarity of the final Urdu version and then examined it for cultural and religious appropriateness. Subsequently, two new translators, also fluent in English and Urdu and not involved in the first stage of the process, translated the final Urdu version back into English. The next step involved a bilingual expert comparing the two back-translations to identify inconsistencies or errors. The translators and the study authors discussed all discrepancies to ensure that the Urdu version faithfully reflected the original English text. To assess the reliability and accuracy of the translation, we used two popular statistical tools for measuring consensus among several raters: the intraclass correlation coefficient (ICC) [19] and Cohen’s kappa coefficient [20]. To gauge the degree of consensus between two translators, the kappa coefficient (or ICC) was computed for every item on the questionnaire. The results demonstrated that the translations were consistent, with the Cohen’s kappa coefficient and the ICC both surpassing 0.99. Pilot testing was the last step toward ensuring that the Urdu version was easy to understand and unambiguous. At this stage, the research team administered the questionnaire to 25 Urdu speakers. No difficulties were encountered, so this version was used for the main study.

For the purpose of this study, ghosting was defined as “abruptly ending the continuity of a relationship without providing a reason.” The study translated and validated the English-language GQ into Urdu. The translated version comprises eight items and uses a 5-point Likert scale, i.e., never (1), rarely (2), sometimes (3), often (4), and always (5). The higher the score, the more experience of ghosting or having been ghosted.

### 2.2. Participants

The study included 540 participants, of which 200 (37%) were male and 340 (63%) were female. In terms of occupation, the majority were students (n = 469, 87%); n = 63 (12%) were employed and n = 6 (1%) were unemployed. Regarding marital status, 461 (85%) were single and 79 (15%) were married. The mean age of the participants was 22.37 (SD = 6.22), with a range between 14 and 56. The mean education level was 14.04 (SD = 2.24) years of schooling.

### 2.3. Data Collection

The study participants were recruited at government and public sector universities in Islamabad, Pakistan. Before taking part in the study, all provided informed consent. The commonly accepted guidelines for social surveys recommend from 5 to 10 participants per questionnaire item [21]. The current study involved 540 participants in total. There was a two-week interval between the original administration of the questionnaire and the second administration to a subset of participants (n = 100), whose responses were used to assess test–retest reliability. The statistical power of the factor analysis and other psychometric analyses utilized in this study was approximately 90%.

### 2.4. Ethics

All steps of the research were conducted in accordance with the 1964 Helsinki Declaration and subsequent updates. Ethical approval for the research came from Pakistan’s COMSATS University Islamabad’s Department of Humanities. All the participants in the study freely participated.

### 2.5. Statistical Analyses

All the statistical analyses, including computation and visualization, were conducted using the R Statistical Foundation (version 4.3.2). A *p* value of less than or equal to 0.05 was used to evaluate significance. Descriptive statistics, including standard deviation and mean, were used to describe and summarize the results.

The English and Urdu versions of the sample underwent rigorous evaluation to assess their adequacy for sampling [22]. The Kaiser–Meyer–Olkin (KMO) test is a statistical measure used to assess the sampling adequacy in a factor analysis [22]. It determines whether the data collected from a particular sample are suitable for conducting further analyses [22]. Thus, both the English and Urdu versions of the sample were subjected to the KMO test [22]. The KMO value ranges between 0 and 1, with higher values indicating a better sampling adequacy [22]. Additionally, Bartlett’s test of sphericity was performed to assess whether the variables in the data were correlated [22]. This test helps to determine whether the data are suitable for conducting factor analysis [22]. In this study, Bartlett’s test confirmed that both the English and Urdu versions had sufficient data (*p* < 0.001), indicating a significant correlation between the variables [22].

For this study, we evaluated the internal consistency of the Urdu GQ using Cronbach’s alpha and McDonald’s omega. A reliability level higher than 0.75 was considered as acceptable [22]. To assess test–retest reliability, a random subsample of 100 participants completed the Urdu GQ twice, with a 2-week interval between administrations. Intraclass correlation (ICC) analysis was used to evaluate the temporal stability of the scores. We used confirmatory factor analysis (CFA) to evaluate the factor structure of the Urdu GQ. Based on the one-factor model, it was determined that all the items assessed the same construct as the original English-language version. Structural equation modeling (SEM) was used to conduct the CFA using the maximum likelihood estimation method. Fit indices such as the comparative fit index (CFI), Tucker–Lewis index (TLI), root mean square error of approximation (RMSEA), and standardized root mean square residual (SRMR) were used to measure the goodness of fit of the models [22]. For the CFI and TLI, values above 0.90 indicate an acceptable fit and values above 0.95 indicate an excellent fit [22]. For RMSEA, values less than 0.08 suggest a reasonable fit and values less than 0.05 suggest a close fit [22]. SRMR values less than 0.08 are considered to be a good fit. In addition to these overall model fit indices, individual factor loadings above 0.4 were considered to be satisfactory [22].

## 3. Results

Both the English and Urdu versions of the GQ were determined to show high levels of reliability by Cronbach’s alpha and McDonald’s omega (Table 1; English Version: α = 0.907, ω = 0.910; Urdu Version: α = 0.913, ω = 0.915). The ICC analysis showed significant test–retest reliability, as indicated by the significant intraclass correlation coefficient (ICC = 0.960; *p* < 0.001) among the 100 participants who re-responded to the questionnaire two weeks later.

The English (KMO = 0.914) and Urdu (KMO = 0.918) versions of the sample were deemed to have adequate sampling according to Kaiser–Meyer–Olkin’s values [23]. Both versions were found to have sufficient data (*p* < 0.001) according to Bartlett’s test of sphericity [24,25].

A confirmatory factor analysis (CFA) was conducted to evaluate the one-factor structure of the Urdu GQ. The hypothesized single-factor model showed a good fit with the data, as indicated by the CFI (0.991), TLI (0.987), NFI (0.983), and other incremental fit indices exceeding 0.95. The RMSEA of 0.045 and SRMR of 0.018 were below the recommended cutoffs, and the significant model chi-square (χ^2^(20) = 41.95, *p* = 0.003) could be attributed to the large sample size. All the factor loadings were statistically significant and ranged from 0.812 to 1.070. The average variance extracted was 0.572, satisfying the recommended threshold for convergent validity. The unidimensional factor structure was supported, with high loadings suggesting that the scale items effectively measured the underlying construct of ghosting. The CFA provides empirical validation of the factorial validity and psychometric soundness of the Urdu GQ. Table 2 provides the CFA results for the GQ Urdu (n = 504).

The convergent validity of the GQ-U was confirmed by the strong correlation between the Urdu and English versions of the GQ (Table 3; r = 0.947; *p* < 0.001). Correlations between ghosting and age (r = 0.386; *p* < 0.001) and education (r = 0.218; *p* < 0.001) were also found to be statistically significant. See Table 3.

## 4. Discussion

The translation of the GQ into Urdu will allow researchers in South Asia, especially in Pakistan and India, to investigate the frequency of ghosting in new sociocultural settings. The standardized translation and validation process of the present study guarantees that the Urdu version is culturally and linguistically appropriate. The English and Urdu versions of the GQ were found to be reliable and valid. The reliability of the instrument is supported by a high internal consistency, as confirmed by Cronbach’s alpha and McDonald’s omega. By confirming the similarity of the questionnaire to its original English version, the Urdu version lays solid groundwork for its use in subsequent studies. The significant test–retest reliability highlights the stability of the responses over time.

This study adds to the body of knowledge on ghosting by introducing a valid measure into a South Asian cultural setting. An in-depth understanding of the psychological basis of ghosting behaviors is provided by the theoretical frameworks utilized to construct the questionnaire. Using this tool, future researchers can investigate the extent to which ghosting is prevalent in various cultural settings across a large swath of the world and compare the variables that contribute to this modern interpersonal phenomenon. The GQ is the only existing tool for assessing this construct. Due to this limitation, we were unable to examine convergence with unrelated instruments, as would have been ideal. To partially compensate for this lack, we evaluated the relationship between the Urdu University translation score and the original English-language GQ score. The correlation was excellent.

### Strengths and Limitations

One strength of this paper is the extensive process undertaken for validating and translating the GQ into Urdu. This study ensures that the instrument is suitable for use in South Asian contexts, especially in Pakistan and India, by taking not only language, but also cultural aspects into account. Future research on ghosting in diverse sociocultural settings will be made easier by this effort, which will also improve the cross-cultural applicability of the GQ.

Even though this study adds to our knowledge of ghosting behaviors, we must be mindful of its limitations. One concern is that responses may be driven by social desirability bias. Secondly, caution is needed when projecting the results to a larger Urdu-speaking population, as the study was conducted in Islamabad, Pakistan, which may not be representative of the diverse Urdu cultural contexts of South Asia. Thirdly, ghosting, a currently prevalent behavior, is likely to evolve, with its expression and consequences changing over time as the use of technology advances. Finally, the current validation study involved a homogeneous sample recruited from a university population. While the convenience sampling of students enabled the initial evaluation of the Urdu GQ, this approach restricts the generalizability of the findings. The sample lacked diversity in terms of age, different relationship categories, and other demographics that may influence experiences with ghosting. Measurement invariance across different subgroups needs to be examined in future research. Wider sampling from the community would provide a stronger evaluation of the factor structure and reliability of the Urdu GQ. This tool requires further validation using broader recruitment strategies to capture a more representative cross-section of the population. Although the student sample provides preliminary evidence of the questionnaire’s adequacy, confirmation of its psychometric properties and utility for measuring ghosting experiences in the general public is needed. Ensuring cultural and demographic inclusiveness should be a priority.

The implications of the GQ in research and clinical practice are many. First, it provides a common measurement tool for researchers across Pakistan and India to systematically examine the prevalence, predictors, and impacts of ghosting in the region’s diverse populations. Both quantitative surveys and qualitative studies could employ the questionnaire to uncover previously unexplored cultural patterns and insights into ghosting behaviors and ghostee responses. Second, the availability of the Urdu questionnaire also opens up possibilities for evaluating interventions aimed at reducing ghosting or mitigating its negative effects. Programs teaching healthier relationship communication skills to the youth could use the questionnaire as a pre–post assessment of their impact. The scale could also assess the outcomes of public awareness campaigns or online platform policies designed to curb ghosting practices. Third, mental health professionals in the region now have access to a validated tool for helping clients cope with ghosting experiences in counseling contexts. The questionnaire provides a framework for therapists to better understand and address their clients’ feelings and behaviors in response to ghosting.

## 5. Conclusions

In this study, we addressed the lack of a psychological tool for assessing ghosting behavior in Urdu. We translated, adapted, and validated the GQ for Urdu speakers, a large population in India and Pakistan. Our translation process involved forward and backward techniques, and the psychometric analyses included confirmatory factor analysis, convergent validity, internal consistency, test–retest reliability, and goodness of fit. The Urdu version of the GQ demonstrated excellent initial and test–retest reliability. The confirmatory factor analysis revealed a one-factor structure aligning with the original English version of the GQ. The Urdu version of the GQ proved to be a reliable and valid tool for evaluating ghosting behavior among Urdu speakers. This adaptation facilitates research and clinical assessments in Pakistan and India, aiding the study of ghosting in a very largely populated world region. Future research needs to explore cultural variations in ghosting behavior among Urdu-speaking individuals and contribute to the validation of the questionnaire in clinical settings.

## Figures and Tables

**Table 1:** Descriptive statistics and reliability analyses (n = 504).

Variable	N	Items	α	ω	M	SD	%	Range		Skewness	Kurtosis
								Potential	Actual		
GQ (English)		8	0.907	0.910	21.804	7.561	54.509	8–40	8–40	0.282	−0.604
GQ (Urdu)		8	0.913	0.915	21.524	7.752	53.810	8–40	8–40	0.263	−0.645

Notes: GQ = Ghosting Questionnaire. N = Number of participants; α = Cronbach’s alpha; ω = McDonald’s omega; M = mean; SD = standard deviation; Kaiser–Meyer–Olkin measure of sample adequacy = 0.914 (English) and 0.918 (Urdu); *p* value for Bartlett’s test of sphericity = 0.001 (both languages); %= prevalence of being ghosted by the participants estimated at a GQ score > 30.

**Table 2:** Confirmatory factor structure for the GQ Urdu (n = 504).

Item No.	Item *	Loadings	z, *p* Value
1	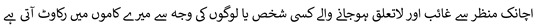	0.81	17.925, <0.001
2	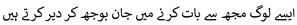	0.89	21.239, <0.001
3	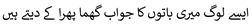	0.87	19.585, <0.001
4	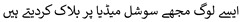	0.90	18.191, <0.001
5	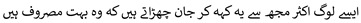	0.99	22.119, <0.001
6	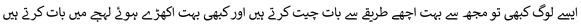	0.97	21.211, <0.001
7	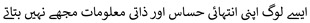	0.88	18.276, <0.001
8	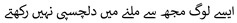	0.99	22.337, <0.001

Notes: * Items in Urdu, the original English language items are: 1. Did you get stood up or had your plans canceled by them without being told beforehand. 2. Their reply/response messages are delayed. 3. Their reply/response messages are confusing and vague. 4. Have you been blocked or deleted from their social media apps or messaging apps. 5. The phrase “I’m busy” is always used in their communications. 6. Their interest in you is inconsistent sometimes very engaged, sometimes completely uninterested. 7. They don’t share personal information about themselves with you. 8. They are not interested in meeting. Confirmatory factor structure estimated using maximum likelihood extraction (MLE). Fitness measures Χ^2^, (df) = 41.95, 20, Comparative Fit Index (CFI) = 0.991, Tucker–Lewis Index (TLI) = 0.987, Bentler–Bonett Nonnormed Fit Index (NNFI) = 0.987, Bentler–Bonett Normed Fit Index (NFI) = 0.983, Parsimony Normed Fit Index (PNFI) = 0.702, Bollen’s Relative Fit Index (RFI) = 0.976, Bollen’s Incremental Fit Index (IFI) = 0.991, Relative Noncentrality Index (RNI) = 0.991, Log-likelihood = −5826.861, Number of free parameters = 24, Akaike (AIC) = 11,701.721, Bayesian (BIC) = 11,804.719, Sample-size adjusted Bayesian (SSABIC) = 11,728.534, Root mean square error of approximation (RMSEA) = 0.045, RMSEA 90% CI lower bound = 0.026, RMSEA 90% CI upper bound = 0.064, RMSEA *p* value = 0.638, Standardized root mean square residual (SRMR) = 0.018, Hoelter’s critical N (α = 0.05) = 405.346, Hoelter’s critical N (α = 0.01) = 484.59, Goodness-of-fit index (GFI) = 0.994, McDonald fit index (MFI) = 0.98, Expected cross validation index (ECVI) = 0.167.

**Table 3:** Correlations between the GQ (Urdu) score or the GQ (English) score and selected sociodemographic variables (n = 504).

	GQ (Urdu)	Age	Education
GQ (English)	0.947 *	0.396 *	0.239 *
Age	0.386 *		
Education	0.218 *		

Notes: GQ = Ghosting Questionnaire. * Correlation is significant at the 0.01 level (2-tailed).

## Data Availability

The corresponding author can provide the data upon request.
